# Hunting of roe deer and wild boar in Germany: Is non-lead ammunition suitable for hunting?

**DOI:** 10.1371/journal.pone.0185029

**Published:** 2017-09-19

**Authors:** Annett Martin, Carl Gremse, Thomas Selhorst, Niels Bandick, Christine Müller-Graf, Matthias Greiner, Monika Lahrssen-Wiederholt

**Affiliations:** 1 Department Exposure, Federal Institute for Risk Assessment (BfR), Berlin, Germany; 2 Department Safety in the Food Chain, Federal Institute for Risk Assessment (BfR), Berlin, Germany; 3 Department Biological Safety, Federal Institute for Risk Assessment (BfR), Berlin, Germany; Universita degli Studi di Sassari, ITALY

## Abstract

**Background:**

Non-lead hunting ammunition is an alternative to bullets that contain lead. The use of lead ammunition can result in severe contamination of game meat, thus posing a health risk to consumers. With any kind of ammunition for hunting, the terminal effectiveness of bullets is an animal welfare issue. Doubts about the effectiveness of non-lead bullets for a humane kill of game animals in hunting have been discussed. The length of the escape distance after the shot has been used previously as an indicator for bullet performance.

**Objective:**

The object of this study was to determine how the bullet material (lead or non-lead) influences the observed escape distances.

**Methods:**

1,234 records of the shooting of roe deer (*Capreolus capreolus*) and 825 records of the shooting of wild boar (*Sus scrofa*) were evaluated. As the bullet material cannot be regarded as the sole cause of variability of escape distances, interactions of other potential influencing variables like shot placement, shooting distance, were analyzed using conditional regression trees and two-part hurdle models.

**Results:**

The length of the escape distance is not influenced by the use of lead or non-lead ammunition with either roe deer or wild boar. With roe deer, the length of the escape distance is influenced significantly by the shot placement and the type of hunting. Increasing shooting distances increased the length of the escape distance. With wild boar, shot placement and the age of the animals were found to be a significant influencing factor on the length of the escape distance.

**Conclusions:**

The length of the escape distance can be used as an indicator for adequate bullet effectiveness for humane killings of game animals in hunting.Non-lead bullets already exist which have an equally reliable killing effect as lead bullets.

## Introduction

The replacement of lead-ammunition with non-lead ammunition for hunting of game animals is presently discussed. Game meat shows comparatively high concentrations of lead [1, 2].

Increased lead levels were detected in the blood of people frequently consuming game meat (especially in hunting households) [3–5]. Since no safe threshold level for lead can be recommended anymore [1], steps have to be taken if possible to minimize lead intake through food consumption. Furthermore, various authors [6–9] also make reference to the contamination of ecosystems through the use of lead ammunition. Animals shot with lead ammunition which die subsequently without being found are often the cause of lead poisoning in waterbirds and birds of prey [6–9].

Therefore, the reduction of the lead exposure of the consumer and to the environment through the substitution of lead with non-lead ammunition is recommended, but has to be brought into balance with the killing effect of bullets as prescribed by animal welfare regulations. According to Article 1 of the German Animal Welfare Act, animals must be protected from unnecessary pain, suffering and damage [10]. Currently, there is an ongoing debate about the suitability of non-lead ammunition. It must be guaranteed that game animals are killed quickly and reliably with bullets of any construction.

Some authors expressed doubt over the killing efficiency of non-lead ammunition in practical hunting [11–13].

Tests were conducted in various countries to assess the killing effect of lead and non-lead ammunition in day-to-day hunting practice with contradictory results. Some studies showed no difference between lead and non-lead ammunition in terms of escape distance.

In Scotland, comparisons were made of the killing effect of red and roe deer shot with lead or copper ammunition [14]. Of 34 red deer shot with lead bullets, 79% (27 animals) of the shots were evaluated as successful (good killing effect). Sixty-two red deer were killed with bullets made from copper. Of these, 77% (48 animals) of the shots were evaluated as successful. The results of this study suggest that there is no difference in the killing effect of lead and non-lead ammunition.

In Austria, records of 1,231 killings of various species by professional hunters were analysed taking a total of 55 variables into account in the statistical analysis in addition to the bullet material. The game species had the greatest effect on the length of the escape distance. The placement of the shot and weight class of the animals were also significant along with their interactions. The bullet material had no influence on the length of the escape distance [15].

In a study conducted in Germany with copper ammunition [16], 95% of the game animals (n = 260) were killed with a single shot. For 48% of these instances, the escape distance was 0 meters. Animals, which ran after being shot, covered on average an escape distance of 14 meters. Of the 12 participating hunters, eight were convinced that copper bullets are just as suitable as conventional lead ammunition. Four hunters were of the opinion that copper ammunition is more suitable [16].

To compare the terminal effectiveness of non-lead ammunition with conventional lead ammunition, 11,371 data records on the killing of ungulates in Germany in relation to the escape distance estimated by the hunters were evaluated. The evaluation of the data on the effect of the projectile (escape distance) and effectiveness of the projectiles in the lab tests showed no differences in the escape distances of animals shot with lead and non-lead ammunition when minimum terminal ballistic requirements were met [17].

No significant difference between lead and non-lead ammunition was found in a study looking at the extent of the injuries–rather than escape distance—on the basis of the diameter and maximum cross-sectional surface of the wounds of hoofed animals shot in the thorax [18].

Other studies found longer escape distances when using non-lead ammunition.

A Danish study [19] compared the escape distances of roe deer shot with non-lead and lead bullets. When non-lead ammunition was used at shooting distances over 100 m the escape distances significantly increased. However, this finding did not change the practice of hunting in Denmark [19].

The German Hunting Association (DJV) conducted a survey among 1,688 hunters, either hunting with lead or lead-containing ammunition. Of the participants who used non-lead bullets, 36% changed back to lead ammunition. The most commonly mentioned reason for doing so was the poor killing effect [13]. However, the method for determining the killing effect was not described in any detail.

In 2011, the Association of German Professional Hunters participated in a comparison of the use of lead and non-lead ammunition. Without exception, the escape distances of animals shot with non-lead ammunition were longer than those of animals shot with ammunition containing lead [12].

Concerns were also put forward regarding the nationwide introduction of non-lead ammunition that the hazard potential of non-lead ammunition could increase for hunt participants due to the altered ricochet behaviour of this type of ammunition. The Federal Ministry of Food and Agriculture (BMEL) commissioned an expert opinion on the ricochet behaviour of lead and non-lead ammunition [20] which reported no significant differences between the two bullet types. It was established in multiple experiments that it is not the material (lead or non-lead) that is important for practical hunting applications in line with animal welfare regulations but rather the design of the bullets [17, 20, 21].

The contradictory results of the influence of the bullet material on the killing effect lead to considerable uncertainty among many hunters and are the object of controversial discussion with regards to regulations. For this reason, the escape distances of roe deer and wild boar were analysed with data from the research project “Safety of game meat obtained through hunting” [22]–with detailed records on the killing—in order to determine the influencing factors of the length of the escape distance. Escape distance was used as the measure for the killing efficiency of bullets on game animal and was estimated by the hunters. The hypothesis that the length of the escape distance of roe deer and wild boar is independent of the bullet material (lead and non-lead) was to be tested. As the bullet material (lead vs non-lead) cannot be regarded as the sole cause of the varying escape distance lengths, interactions with the location of the shot placement, hunting method, shooting distance, bullet type and age and sex of the animals were also examined.

## Material and methods

### Questionnaire and field observations

Data were collected for the research project “Safety of game meat obtained through hunting” [23, 24] a large project initiated by the Federal Ministry of Food and Agriculture to obtain knowledge-based information on use of lead or non-lead ammunition.

The number of samples required was determined statistically prior to the start of the project and targeted to estimate the lead contents in game meat. The sampling plan [25] included species (roe deer, wild boar), bullet material (lead, non-lead) and region (6 regions with different soil contamination with lead). Overall, 1,254 roe deer were shot and killed in the framework of this study (745 with lead and 509 with non-lead ammunition) and 854 wild boar (514 with lead and 340 with non-lead ammunition).

Various data such as the game tag number (identification number for each game animal), shooter, date and location of kill, hunting district, hunting method, game species with the age, sex and weight of the shot animal, subjectively estimated shooting distance and escape distance, a searching for wounded game with dogs (if applicable) and the bullet used were recorded by the hunters on a standardised sample submission sheet (DOI: 10.17590/20170706-163344).

### Ethics statement

Licensed hunters killed the game analysed in this study during the established hunting season and in accordance with German regulations (German Hunting Act; Bundesjagdgesetz) and best practices. It did not involve any additional killing other than what is carried out in the German forests on a regular and managerial basis (population control). Permission was granted from the respective German Federal States and their hunting authorities.

### Study population

The selection of the two game species roe deer and wild boar was made on the basis of their different foraging behaviour, dominant market shares and occurrence on soils with varying background lead levels. Both species belong to the main game varieties shot in Germany [26].

### Description of the variables

#### Escape distance

The length of the escape distance is based on information provided by the hunters and was estimated in metres.

The escape distance can be considered a key indicator with regard to the killing effect of the bullets. An outright kill is effected when the animals dies immediately after being shot [27]. In this research project we considered animals with an escape distance below 10 meters as being killed on the spot. This means that the animal either dies instantly (escape distance 0 m) or is fatally injured but can still manage to cover a short escape distance (up to 9 m). All escape distances less than 10 m were set to 0. Most hunters estimated escape distances over 10 m in increments of ten, though a few attempted to estimate them more precisely. These distances were rounded off to the nearest increment of ten.

Bullets: With regard to the bullet, the bullet material and type were included in the analyses.

#### Bullet material

**Lead** bullets are produced mainly as semi-jacketed bullets consisting of a core and surrounding jacket. The bullet material is not fully wrapped in the jacket. The core consists of a hard lead alloy and the jacket is made of tombac, a copper-zinc alloy. Upon impact with the animal’s body, the comparably soft lead core disintegrates into tiny splinters which disperse in the animal’s body [28].

**Non-lead** bullets are produced mainly from one material as solid bullets made either of copper or an alloy of copper and zinc [28]. Bullets of this kind can also contain small quantities of lead [29].

#### Bullet types

Depending on how they take effect in the body of the animal, the manufacturers of hunting rifle ammunition differentiate between fragmenting, partially fragmenting and deforming bullets [28]. The bullet types were split into two groups:

1. Fragmenting and partially fragmenting bullets: loss of mass in the target medium is expected
2. Deforming bullets: loss of mass in the target medium is not expected

Details of the calibre were provided by only very few hunters and were therefore not included in the statistical analyses.

#### Shot placement (entry wound)

**Fig 1:**
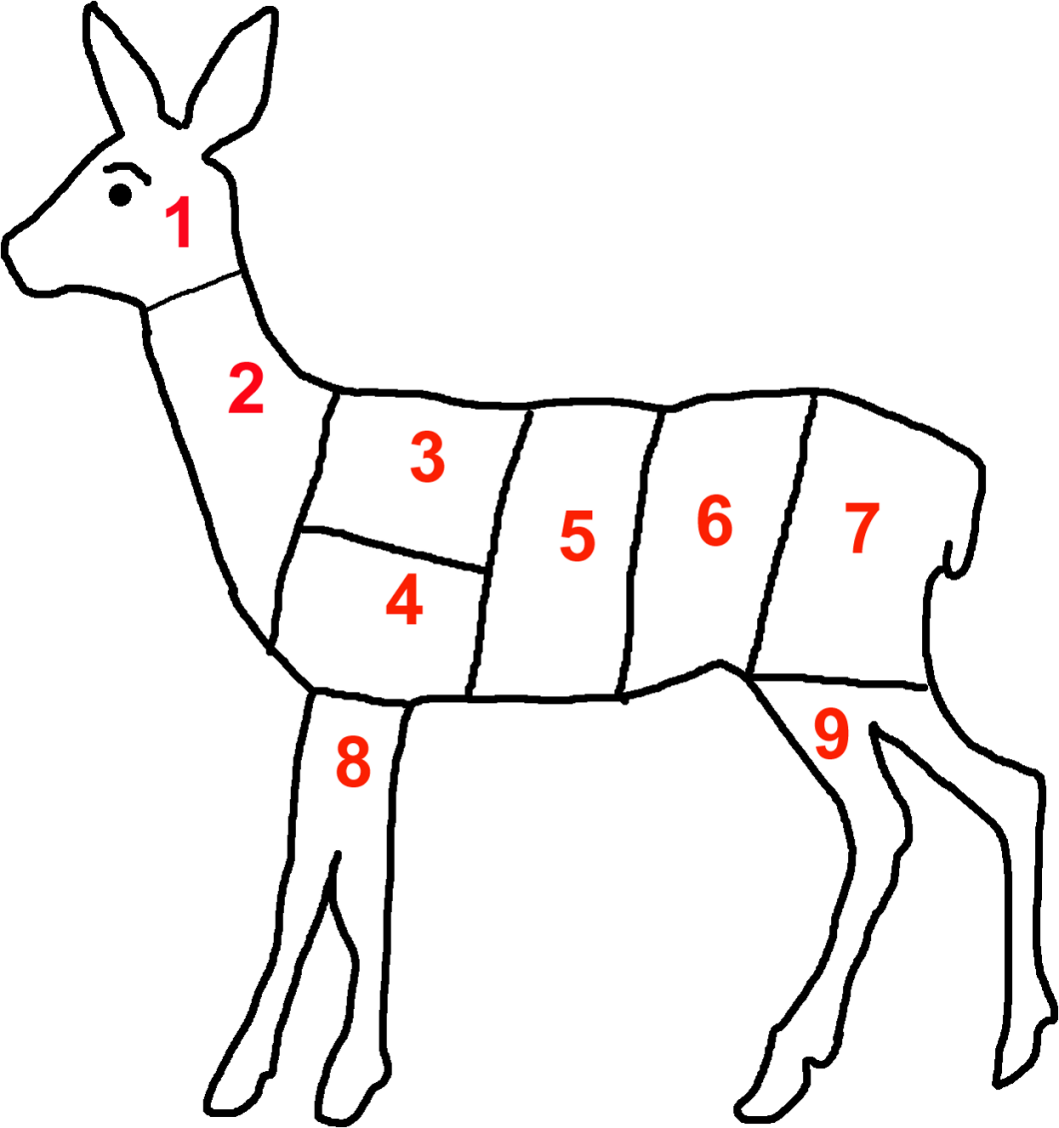
Location of shot placement (entry wound) in roe deer.

The shot placement for the entry wound was given by means of different bullet entry zones. The following bullet entry zones (Fig 1) occurred in different frequencies:

1 head, 2 neck, 3 thorax, upper (lung), 4 thorax, lower (heart, lung), 5 stomach and liver (large intestines), 6 bowels and kidney (small intestines), 7 haunch, 8 forelegs, 9 hind legs. Hits to the head, haunch and forelegs were very seldom or did not occur at all (hind legs).
For statistical analysis 6 categories were formed from the 9 entry zone options: head, neck, thorax (3+4), gastrointestinal tract (5+6), haunch, forelegs.

#### Type of hunt

The game was hunted either from a hide or by stalking or driving. When hunting from a hide, the hunter usually waits on an elevated deer stand until the game moves past. Hunting from a hide is the most common type of hunting in Germany [21, 30, 31].

In contrast, the hunter moves when stalking and tries to get as close to the game as possible [31].

Unlike solo hunting methods like stalking and shooting from a hide, drive hunting is a type of social hunt where a number of hunters hunt together [31]. In a drive hunt, beaters with or without dogs walk steadily through the hunting area in order to set the game animals slowly into motion, while the hunters wait on stands.

#### Age of the animals

The age of the animals was given by the hunters in classes (0, 1, 2–3, 4–5, 6–8, 9–13 years). For statistical analysis these classes were combined into three groups:

1. juveniles (under 1 year)
2. subadults (1- < 2 years)
3. adults (2 years and over)

The weight of the animals was also recorded but was also excluded as it correlated with the age of the animals.

#### Shooting distance

The shooting distances in metres are based on information provided by the hunters.

#### Bone hits

The hunters were able to state whether a bone had been hit or not.

#### Search

The hunters were asked to state whether it was necessary to track down a wounded game animal with a dog.

#### Sex of the animals

The gender of the animals (male/female) was recorded.

A description of all variables is given in S1 Table.

### Statistical analyses

#### Descriptive data analysis

The escape distance, shooting distance, shot placement, hunt method, bullet type, age of animals, any necessary search and bone hits were analysed separately for lead and non-lead ammunition and both game species (roe deer and wild boar). The Chi-square test (χ^2^ test) was carried out to determine whether the categorical variables are evenly distributed with regard to the bullet material. The non-parametric Mann-Whitney U test was used to compare the shooting distance between the two bullet materials. The univariate statistical analyses were conducted with IBM SPSS Statistics version 21.

#### Multivariable data analysis

Selected predictors for escape distance and possible interactions are listed in S1 Table (dichotomous variables bone hits and search excluded). Selection applied the method of conditional inference trees [32]. Conditional inference trees recursively perform univariate splits [32] of the dependent variable based on values of different covariates. Conditional inference trees were constructed using the “ctree” function from the R package Party version 1.0–21 [33] with statistics software R version 3.3.1. These non-parametric regression trees can be used for nominal, ordinal, numerical, censored and multivariate, dependent variables. The *p*-values are given at the junction nodes (split variables) of the tree. These values indicate whether the two groups that result from the split differ significantly in the target variable (escape distance). After construction, the inference trees show the selected predictors and how they interact. The distribution of the individual escape distances is shown in a histogram at the bottom nodes of the tree. One conditional inference tree has been constructed for each species.

Using multivariable hurdle models (hurdle regression), the inference trees were then checked to establish whether the significant interactions are attributable to the actual escape distances (without animals with an escape distance < 10 m) or to the different percentage of animals with escape distances < 10 m compared to animals showing an escape distance of 10 m or more. One of the reasons for selecting these models was the excessive occurrence of escape distances with zero length. Furthermore, over-dispersion conceded with escape distances of 10 m and more. Over-dispersion is existent if the variance exceeds the sample mean. Hurdle models are combined or two-part models [34]. In the first part of the model, the so-called hurdle or binomial part, the probability (p) that the animal died after moving a certain escape distance p(y ≥ 10 m) is estimated against the probability that the animal died on the spot p(y < 10 m). To put it more simply, the hurdle part expresses the likelihood of the animals covering a certain escape distance or not. If there is an excess of zeroes, the value zero is selected as the hurdle. If the hurdle is surmounted, escape distances of y ≥ 10 metres occur. The hurdle part is based on binomial distribution with logit link. The second part of the model is the count data part, which shows how the frequency of certain escape distances (y ≥ 10 m) are influenced by the independent variables (S1 Table). A zero-truncated negative binomial distribution with log link is used here. The count data part therefore focuses on animals that fled.

The overall distribution function f_hurdle_ of random variable Y is described by the following formula (1) [34]:

$$f_{hurdle}(y;x,z,\beta ,\gamma )=\left\{ \begin{array}{ll} \;f_{zero}(0;z,\gamma ) & if\;y=0(y<10m)\\ \left(1-f_{zero}(0;z,\gamma ))\cdot f_{count}(y;x,\beta )/(1-f_{count}(0;x,\beta )\right) & if\;y>0(\geq 10m) \end{array}\right.$$ (1)

where:

z covariates (predictors) for zero process in the hurdle model

*γ* is a parameter for zero process in the hurdle model

x covariates (predictors) for the count model (truncated)

*β* is a parameter for the count model (truncated)

The probability of y < 10 m is given by the distribution function f_zero_ (zero process).

f_count_ is the corresponding distribution function for y = y, y = 10, 20, … (count data part for escape distance y ≥ 10 m).

The model parameters *β* and *γ* are estimated using the maximum likelihood method. The hurdle regression was carried out with the “hurdle” function from the “pscl” package [35] with statistics software R version 3.0.3. The regression coefficients are listed in the results tables with the standard error and the *p*-values for the coefficients.

The significant results of the count data part (escape distances of 10 m and more) are graphically displayed as an empirical cumulative distribution with Microsoft Excel 2010 and R version 3.0.3. An α of 0.05 was defined as the significance level. All *p*-values < α were regarded as significant.

## Results

### Descriptive data analysis

**Fig 2:**
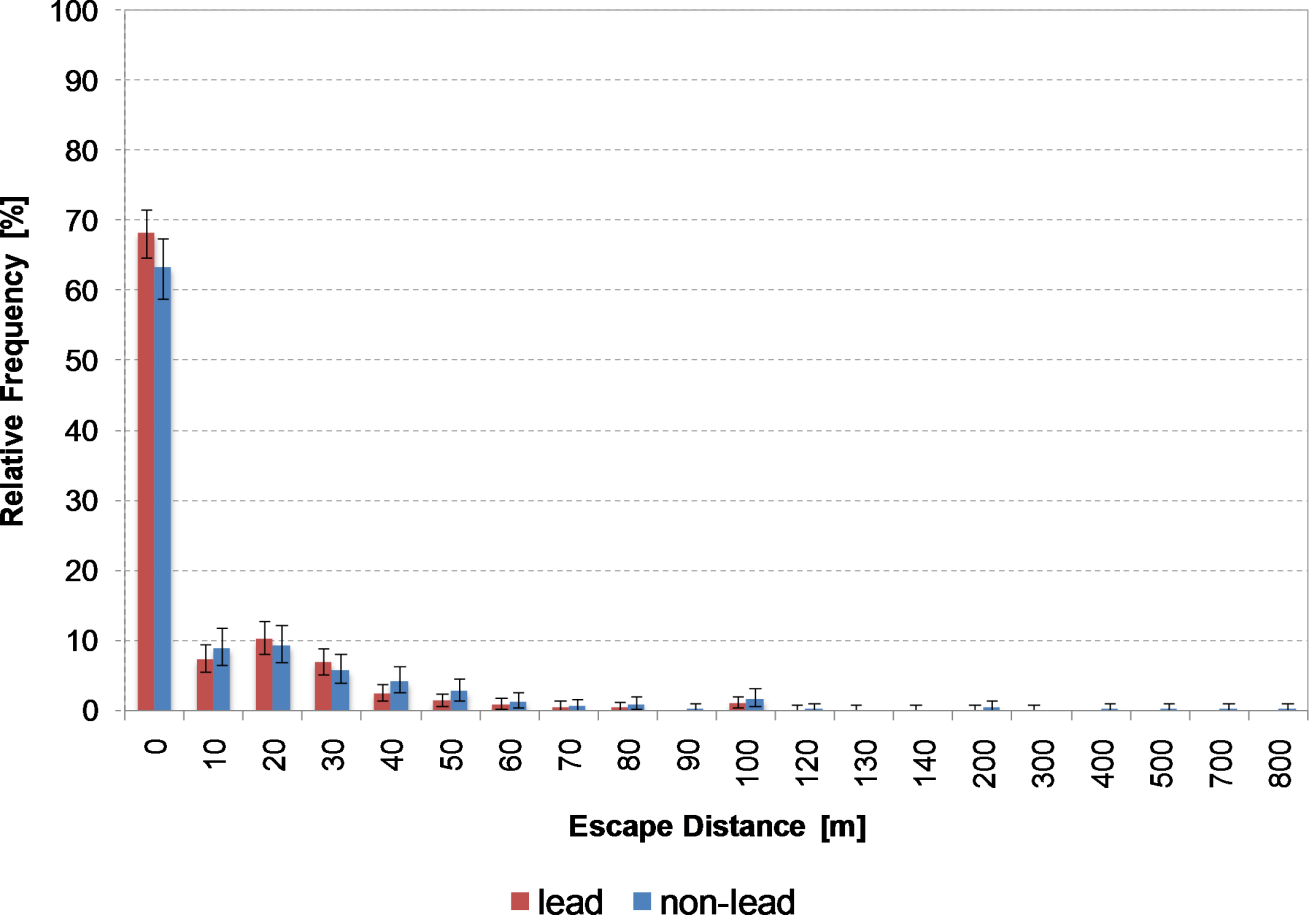
Distribution of escape distances of roe deer with 95% confidence interval. From escape distances above 100 m, ticks on the horizontal x axis are no longer divided into classes of 10 m. Reports of escape distances above 100 m were rare. Escape distances of 0 m also contains distances from 1 to 9 m.

Out of 1,254 shooting records for roe deer, we were able to use 1,234 for statistical analysis, showing that 504 (40.8%) and 730 (59.2%) animals were killed using non-lead and lead ammunition repectively. 20 records contained no details of the escape distance. In the case of wild boar, 825 of the 854 shooting records were included in the analysis, indicating that 333 animals (40.4%) were killed using non-lead and 492 animals (59.6%) using lead ammunition. 29 records contained no details of the escape distance. Fig 2 shows the distribution of the escape distances of roe deer by bullet material. Where non-lead ammunition was used, approx. 63% of the animals remained on the spot (escape distance < 10 m), compared to 68% for lead ammunition. The maximum distance was 800 m (non-lead, one animal).

**Fig 3:**
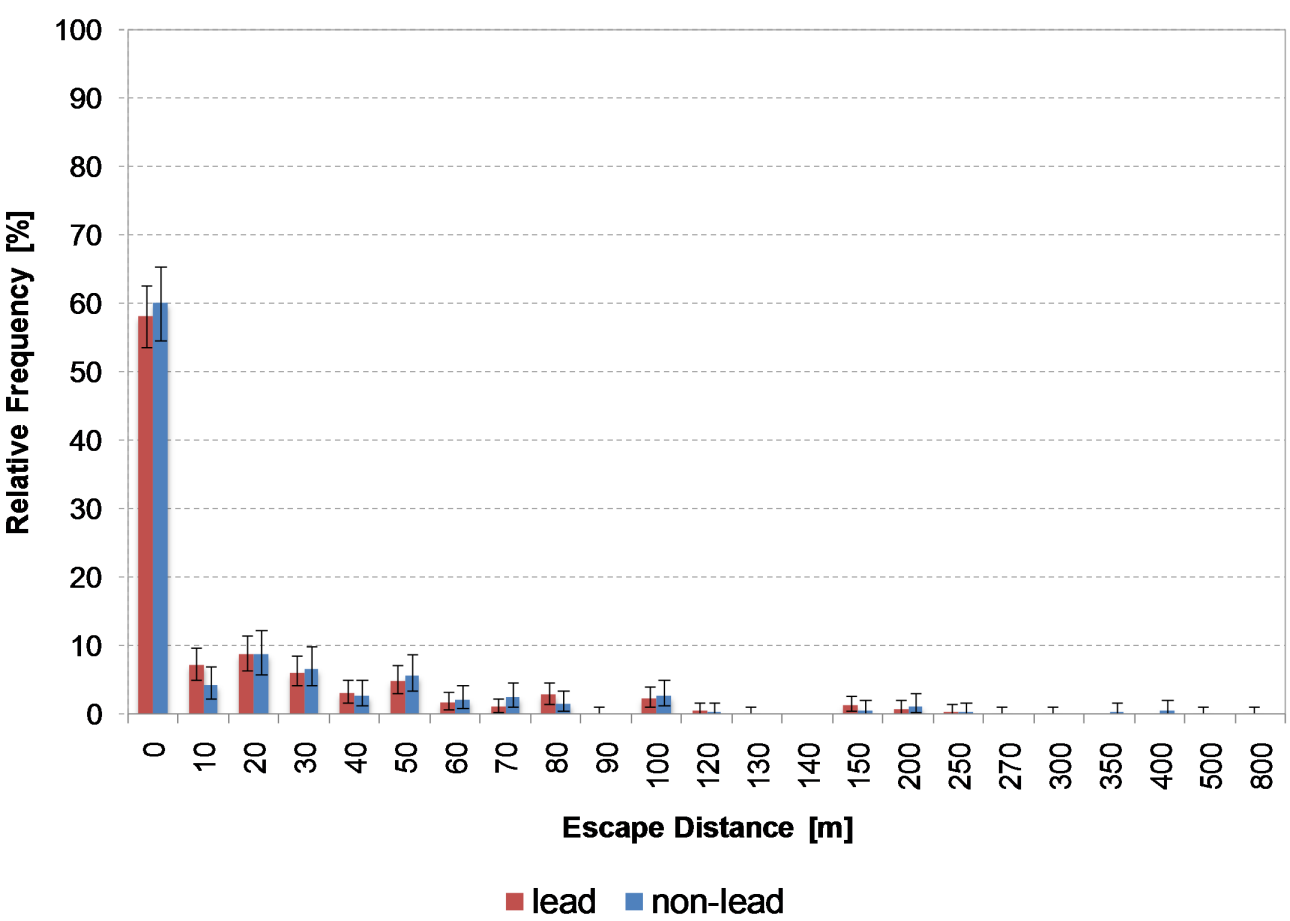
Distribution of escape distances of wild boar with 95% confidence interval. From escape distances above 100 m, the spacings on the horizontal x axis are no longer divided into classes of 10 m. Reports of escape distances above 100 m were rare. Escape distances of 0 m also contains distances from 1 to 9 m.

The escape distances of wild boar (Fig 3) show that 58% of animals shot using non-lead ammunition and 60% of animals hit by lead bullets remained on the spot. One wild boar showed the maximum excape distance of 800 m (lead ammunition used).

When hunting roe deer or wild boar with non-lead ammunition, hunters used 14 deforming bullets and three fragmenting and partially fragmenting bullets from a total of 10 manufacturers. In the case of lead ammunition, a total of eight deforming bullets and 27 fragmenting and partially fragmenting bullets were used from 17 manufacturers. Both in the case of roe deer (χ^2^ = 172.6, df = 1, *p*<0.001) and wild boar (χ^2^ = 72.9, df = 1, *p*<0.001), the lead bullets used were more frequently fragmenting and partially fragmenting bullets than with the non-lead bullets.

**Fig 4:**
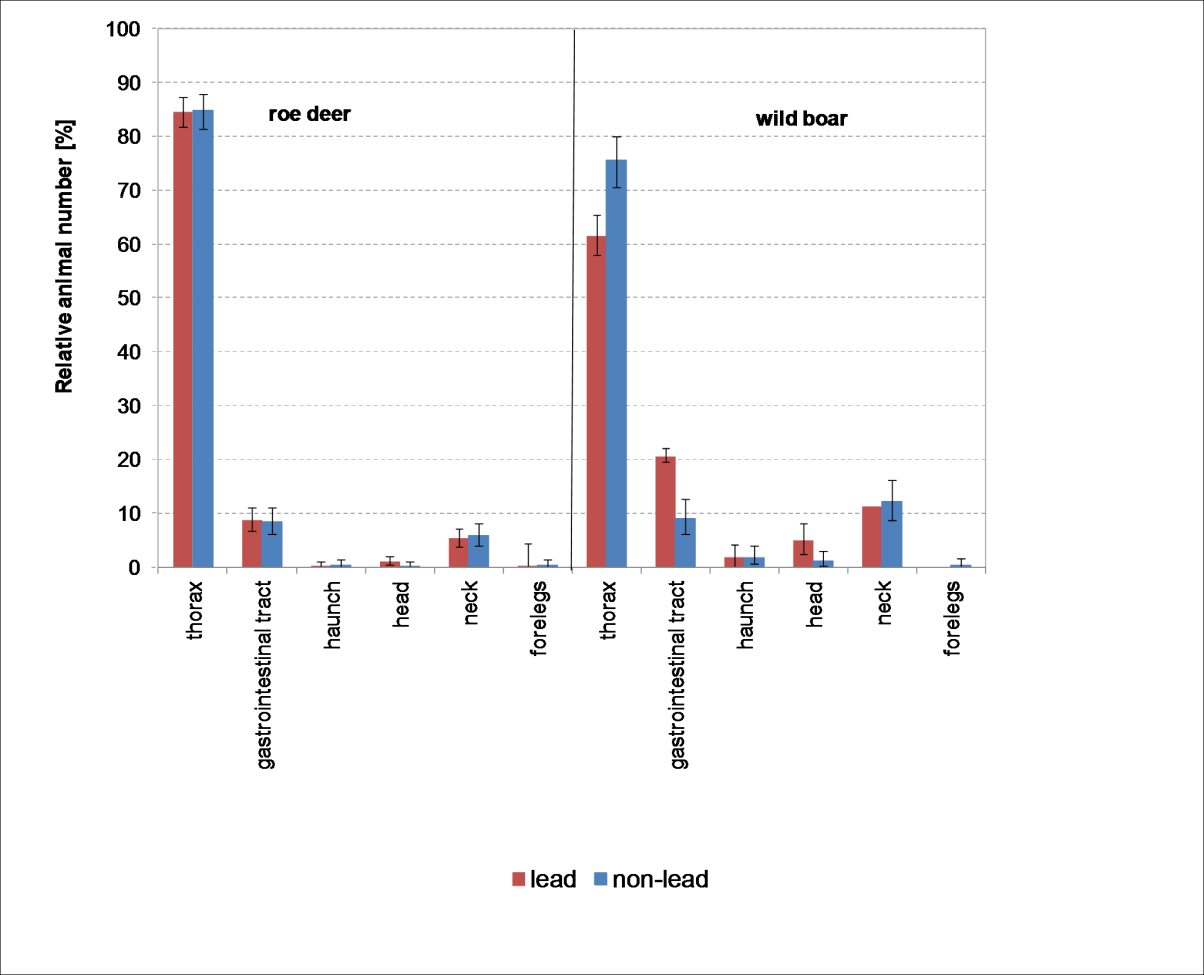
Distribution of shot placement in roe deer and wild boar by bullet material (error bars: 95% CI).

The distribution of shot placement by bullet material (Fig 4) only differed in the case of wild boar (χ^2^ = 31.4, df = 5, *p*<0.001). When lead ammunition was used, the hit was more frequently to the gastrointestinal tract and the head than with non-lead ammunition. Out of a total of 483 animals shot using lead ammunition, the hit was to the gastrointestinal tract in 99 animals (20.5%). Of the 330 animals shot with non-lead bullets, 30 (9.1%) were hit in the gastrointestinal tract. While 5% of animals (24 out of 483) shot with lead bullets were hit in the head, this was the case in 1.2% (4 of 330) of animals shot with non-lead ammunition.

75.5% of the animals (249 of 333) shot with non-lead ammunition were hit in the thorax, compared to 61.5% (297 of 483) shot with lead ammunition.

In the case of roe deer, the distribution of wound locations is the same for both types of bullet material (χ^2^ = 3.8, df = 5, *p* = 0.57, Fig 4).

**Fig 5:**
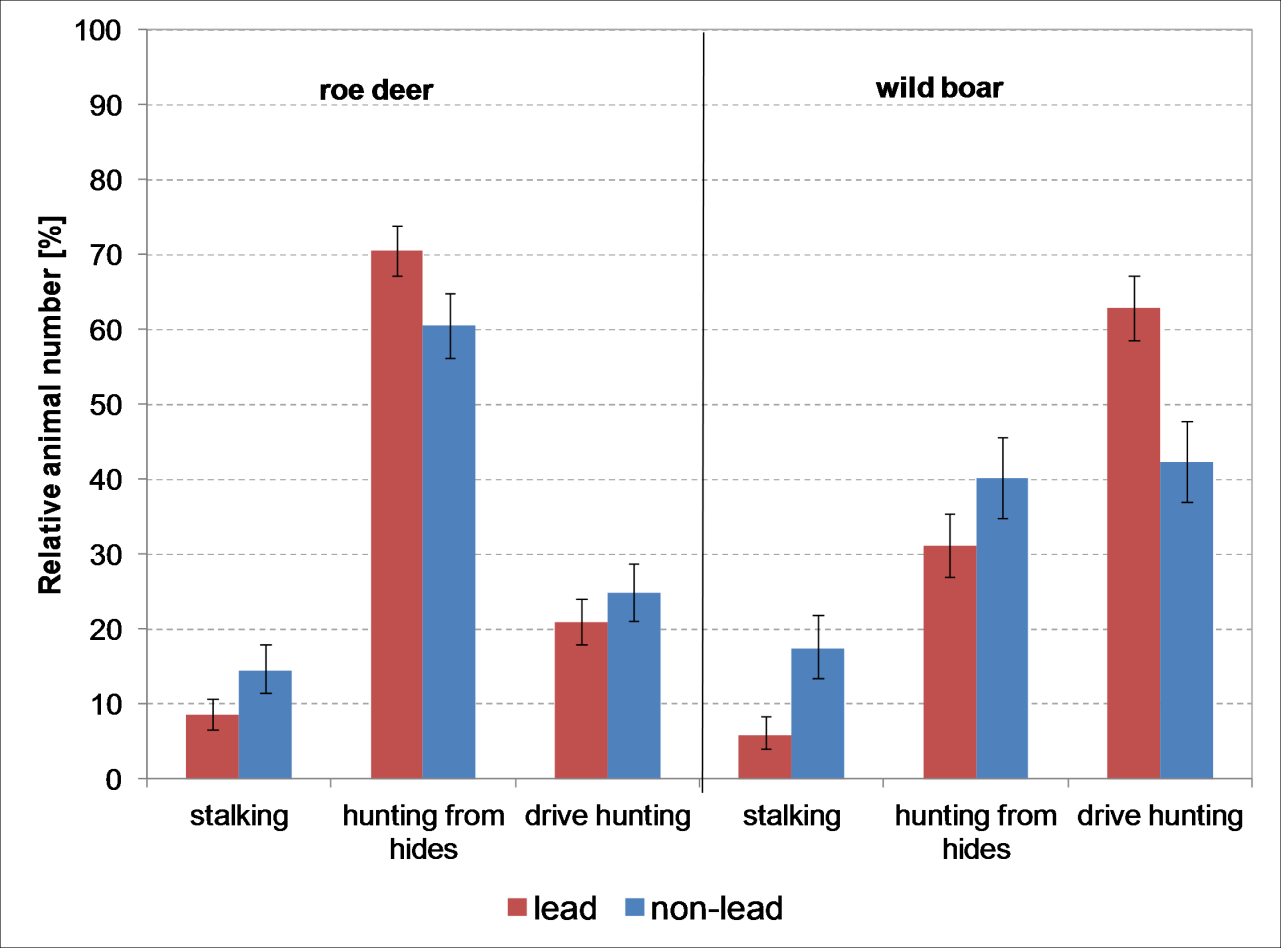
Distribution of hunting methods for roe deer and wild boar by bullet material (error bars: 95% CI).

In the case of roe deer, the distribution of hunting methods (χ^2^ = 16.4, df = 2, *p*<0.001) differed based on bullet material (Fig 5). Where stalking was the method used, roe deer were hunted more frequently with non-lead ammunition (14.4%, 73 of 503 animals) than with lead ammunition (8.5%, 62 of 729 animals). Where hunting was from hides, on the other hand, lead ammunition (70.6%, 515 of 729 animals) was used more frequently than non-lead ammunition (60.6%, 305 of 503 animals). With wild boar, the distribution of hunting methods also differed depending on bullet material (Fig 5) (χ^2^ = 45.3, df = 2, *p*<0.001). With stalking (17.4%, 58 of 333 animals) and hunting from hides (40.2%, 134 of 333 animals), non-lead ammunition was used more frequently than lead ammunition (stalking 5.9%, 29 of 492 animals, and hide 31.1%, 153 of 492 animals). In driven hunts, on the other hand, lead ammunition (63%, 310 of 492 animals) was used more frequently than non-lead ammunition (42.3%, 141 of 333 animals).

**Fig 6:**
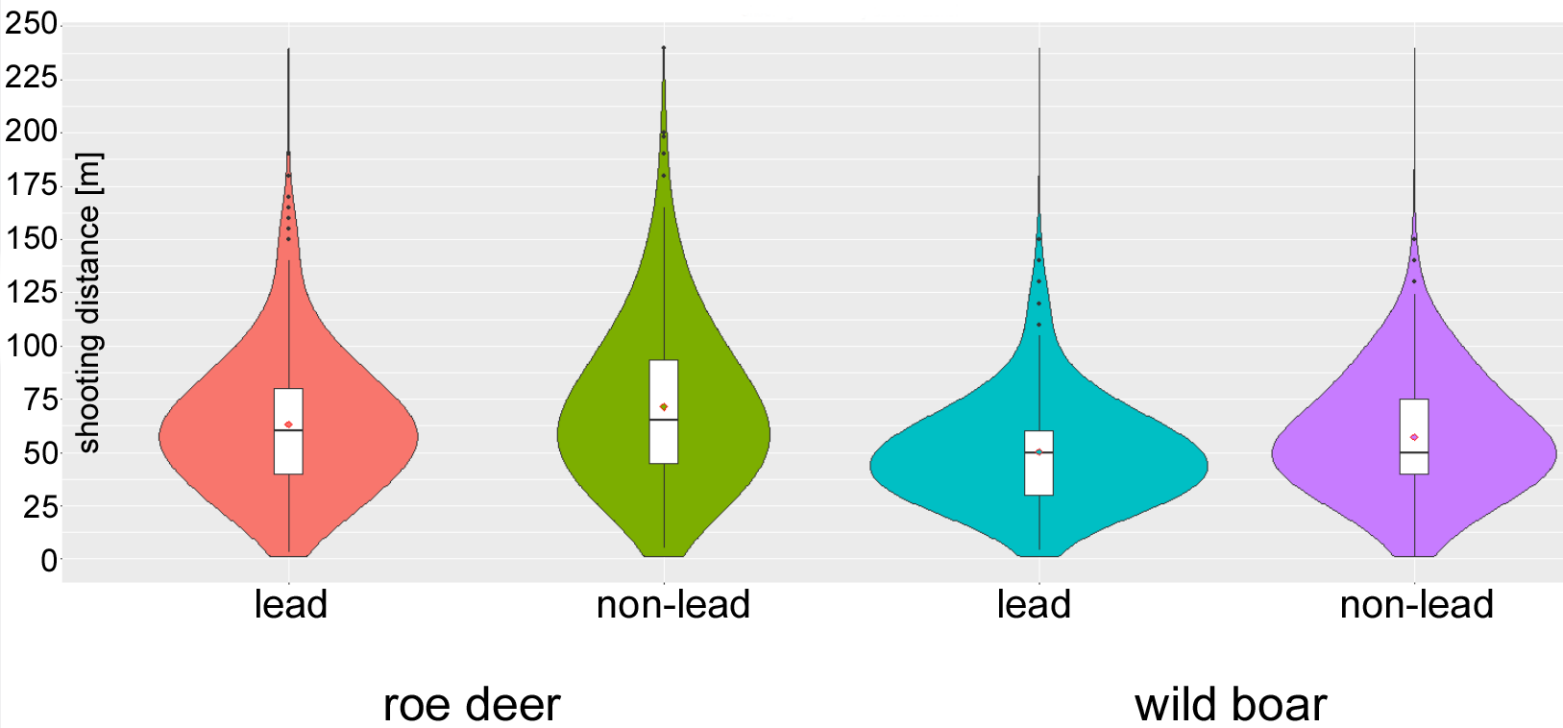
Shooting distance based on bullet material. The violin plot [36] combines the kernel density curve with the box plot. The box plot shows the median and the interquartile spacing. The mean is marked by the point.

The shooting distances for both bullet materials and wildlife species are shown in Fig 6 using violin plots. Compared to lead ammunition, the average shooting distance was higher (U = 163062, *p*<0.001) for roe deer when non-lead ammunition was used (mean 71.5 m vs. 63.4 m; median 65.5 m vs. 60 m; maximum 240 m vs. 190 m; 75th percentile 95 m vs. 80 m). For wild boar hunting, the reported shooting distances with non-lead ammunition (mean 57.3 m, median 50 m, Fig 6) were also higher compared to lead ammunition (mean 51.5 m, median 50 m) U = 67935, *p*<0.001). The maximum shooting distance was 150 m in both cases (non-lead and lead).

In the case of wild boar, bone hits were more frequent when non-lead ammunition (69.1%, 230 of 333) was used (χ^2^ = 14.9, df = 1, *p*<0.001) than with lead ammunition (55.7%, 274 of 492), but no such difference was found with roe deer (χ^2^ = 0.75, df = 1, *p* = 0.38). As the bone hit correlated with the shot placement, this was not included in the multivariable analysis. When roe deer and wild boar were shot in the stomach/intestines, bone hits were significantly more seldom than other hits.

The need to search for wounded game with dogs did not depend on the bullet material with either the roe deer (χ^2^ = 2.8, df = 1, *p* = 0.09) or the wild boar (χ^2^ = 0.20, df = 1, *p* = 0.65). In the case of the roe deer, searches were made for 4.2% (21 of 504) of animals shot with non-lead ammunition, while this was the case with 2.5% (18 of 730) of the animals hit by lead ammunition. 6.3% of the wild boar (21 of 333) shot with non-lead ammunition were searched for, compared to 7.1% of those (35 of 492) shot with lead ammunition. The searching for wounded game was also excluded from the multivariable analysis, as this variable can be seen as a marker for the detailed records on the killing. This variable is also to some extent related to location of shot wound and escape distance and thus already implicitly covered in the data analysis.

Distribution of the sex of the shot animals by bullet material was the same for both types of animals (roe deer: χ^2^ = 3.3, df = 1, *p* = 0.07; wild boar: χ^2^ = 1.4, df = 1, *p* = 0.23).

### Multivariable data analysis

First, inference trees were used to identify predictors and related interactions for the length of the escape distance. The variables and possible interactions between the variables identified using the inference trees were then subjected to closer analysis with hurdle models.

For illustrative purposes, the significant differences in the escape distances (10 m and more) were graphically depicted in the form of a cumulative distribution. As the escape distances of roe deer and wild boar differed, the data were analysed separately for each type of animal.

#### Selection of predictors and related interactions for the escape distance for roe deer

**Fig 7:**
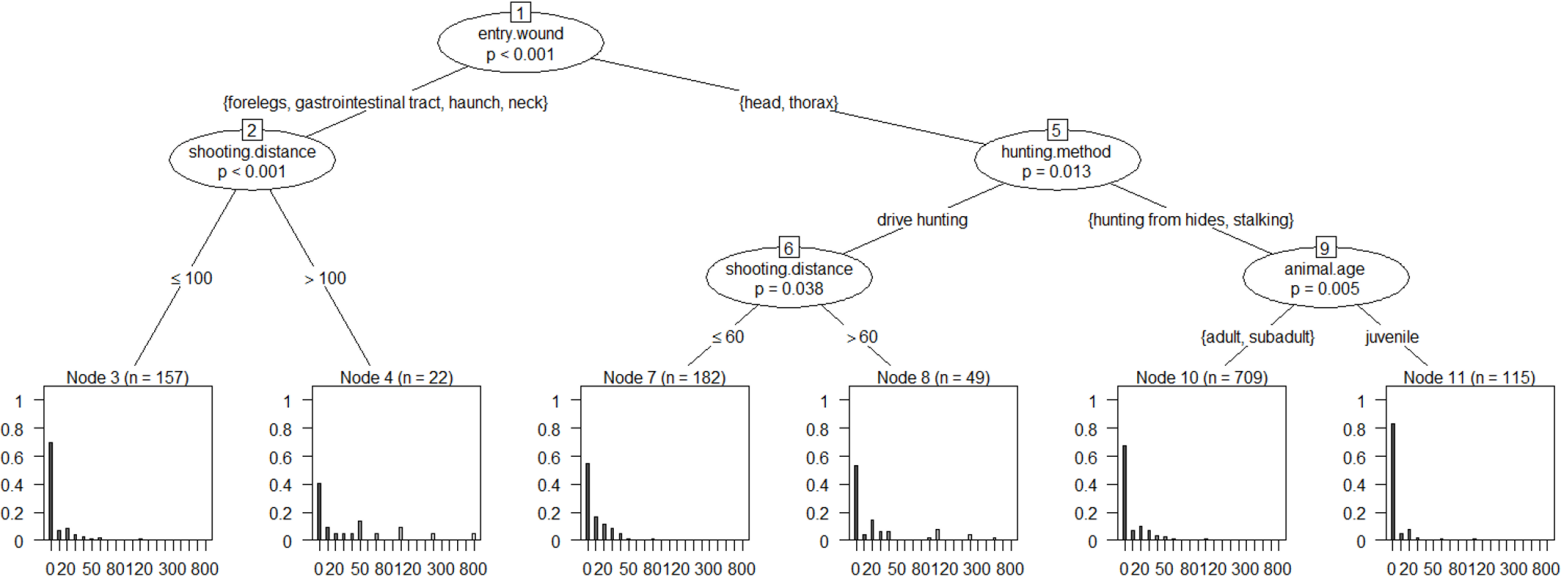
Inference tree for assessment of the factors that influence the escape distance of roe deer (including animals that remained on the spot). The inference tree can be read from top to bottom. The number of the splits (nodes) is shown in the boxes (e.g. 1). Significant variables are indicated at the splits with the significance level in the ellipses. Connections between the nodes show the group distributions. The escape distance is shown as the end node of the inference trees with the help of relative frequencies (with details of the sample size n).

The inference tree in Fig 7 includes roe deer shot on the spot (< 10 m) and animals with escape distances from 10 m upwards.

The variable shot placement (entry wound) is the first selected predictor. The escape distance following hits to the thorax or head differ from those following hits to the forelegs, the gastrointestinal tract, the haunch or the neck (split 1, *p*<0.001).

On condition that roe deer were hit in the forelegs, the gastrointestinal tract, the haunch or the neck there was a significant difference in escape distances depending on shooting distance (split 2, *p*<0.001) of ≤ 100 m or > 100 m.

On condition that the hit was to the thorax or the head, there were significant differences in escape distances between hunting methods driven hunts (split 5, *p* = 0.013) on the one hand and hunting from hides or stalking on the other.

In the case of hits to the thorax or the head, the different shooting distance (≤ 60 m or > 60 m) during driven hunts resulted in different escape distances (split 6, *p* = 0.038).

The escape distances of adult or subadult animals differed from those of animals under one year of age if the animals were killed in stalking hunts or hunting from hides, and if the hit was to the thorax or the head (split 9, *p* = 0.005).

#### Analysis of inference trees using hurdle models for roe deer

The individual splits from Fig 7 are analysed in more detail using multivariable hurdle models in Table 1.

**Table 1:** Hurdle model for roe deer; dependent variable: Escape distance.

	Count model- Zero-truncated poisson			Zero hurdle model Binomial		
	β^1^	Standard error	*p*	β^2^	Standard error	*p*
**Split 1: Comparison entry wound**						
Forelegs, gastrointestinal tract, haunch, neck (intercept)	4.295	0.104		-0.685	0.158	
Thorax, head	-0.857	0.113	***	0.007	0.171	0.97
**Split 2: Condition forelegs, gastrointestinal tract, haunch, neck**						
Shooting distance ≤ 100 m (intercept)	4.066	0.179		-0.850	0.174	
Shooting distance > 100 m	0.671	0.380	0.08	1.218	0.467	**
**Split 5: Condition thorax, head**						
Hunting from hides, stalking (intercept)	3.396	0.045		-0.817	0.076	
Drive hunting	0.154	0.084	0.07	0.614	0.153	***
**Split 6: Condition thorax, head (entry wounds) & drive hunting (hunting method)**						
Shooting distance ≤ 60 m (intercept)	3.196	0.087		-0.262	0.149	
Shooting distance > 60 m	1.111	0.186	***	0.088	0.331	0.79
**Split 9: Condition thorax, head (entry wounds) & hunting from hides, stalking (hunting methods)**						
Juvenile (intercept)	3.185	0.145		-1.630	0.251	
Subadult, adult	0.230	0.151	0.13	0.909	0.263	***

^1^ Escape distances of 10 m and more

^2^ The hurdle part estimates the probability with which the escape distances occur (10 m or more), relative to the probability that no escape distances occur (< 10 m)

**p<0.01

***p<0.001

**Fig 8:**
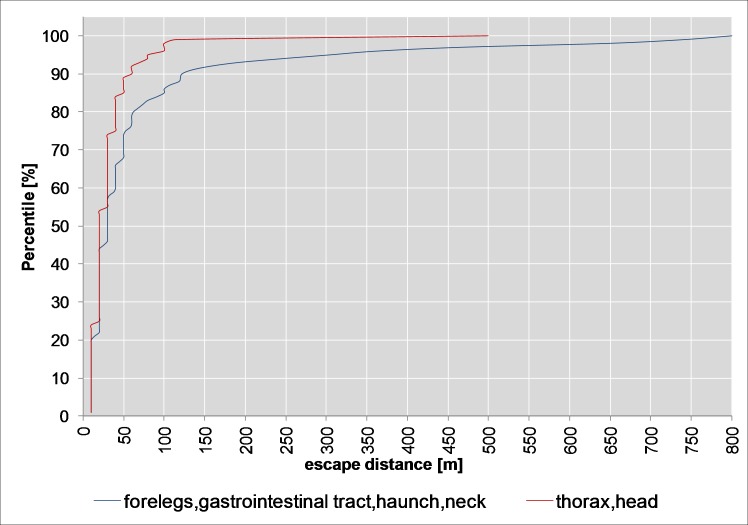
Cumulative density plot for split 1 (roe deer).

The count data part (escape distances of 10 m and more only) of the hurdle model (Table 1, *p*<0.001) and the cumulative probability for split 1 (Fig 8) show that hits to the forelegs, the gastrointestinal tract, the haunch or the neck result in longer escape distances (90th percentile 122 m, median 30 m, max 800 m) than hits to the thorax or head (90th percentile 60 m, median 20 m, max 500 m). But this was not observed when including the animals killed on the spot (binomial part of the hurdle model, Table 1). This means that the percentage of animals with escape distances of 10 m or more is roughly the same as that of animals with an escape distance below 10 m in these hit categories (*p* = 0.97).

When roe deer were hit in the forelegs, the gastrointestinal tract, the haunch or the neck (split 2, Table 1) and the shooting distance was >100 m, only 40% of the animals remained on the spot (escape distance < 10 m). However, with a shooting distance ≤ 100 m, around 70% of the animals remained on the spot. This difference was significant (*p*<0.01, Table 1) when all animals were included in the binomial part of the hurdle model. No differences were observed when only considering the escape distances of the fleeing animals (count data part, split 2, *p* = 0.08).

**Fig 9:**
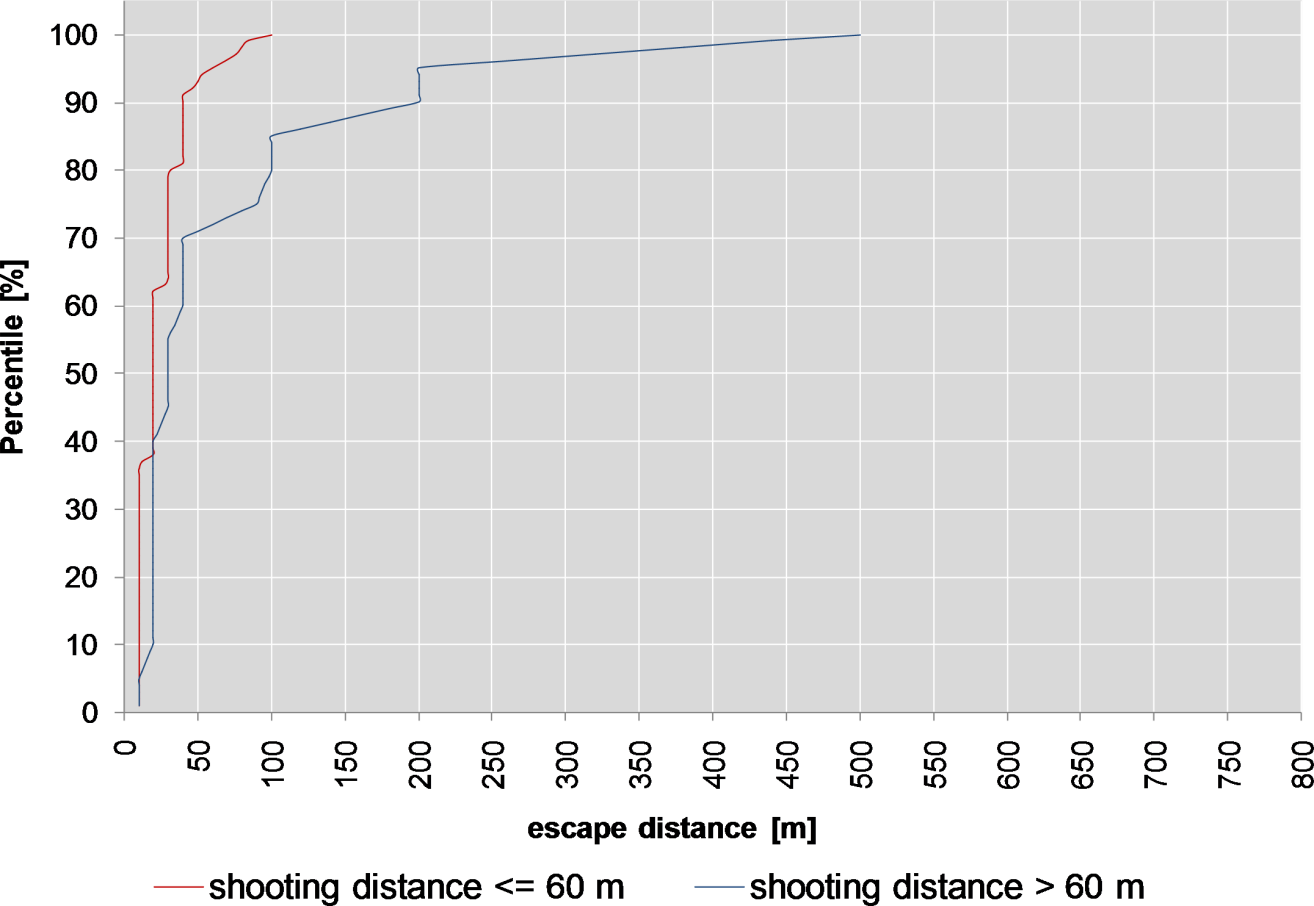
Cumulative density plot for split 6 (roe deer).

In driven hunts, the percentage of animals with escape distances of ≥10 m was higher (47%) than in hunting from hides or stalking (31%). This difference was observed with hits to the thorax or the head (split 5, *p*<0.001, binomial part). In driven hunts and hits to the head or thorax, shooting distances >60 m resulted in longer escape distances than shooting distances ≤60 m (count data part, split 6, *p*<0.001, Table 1). Where roe deer were shot from lower shooting distances (Fig 9, red line), the 90th percentile of the escape distance is approx. 40 m (median 20 m, max 100 m), while with shooting distances >60 m (Fig 9, blue line) this percentile is at 200 m (median 30 m, max 500 m).

When killed by stalking or from a hide and shot in the thorax or head, 67% of adult/ subadult animals remained on the spot, but 82% of the juveniles (binomial part, split 9, *p*<0.001, Table 1). But there was no difference between the escape distances of older and younger animals ≥10 m (count data part, *p* = 0.128, Table 1).

The analysis found that bullet material, the bullet type and the sex of roe deer do not influence the escape distances of roe deer. In other words, the use of lead or non-lead ammunition did not significantly influence the escape distance of roe deer.

#### Selection of predictors and related interactions for the escape distance for wild boar

**Fig 10:**
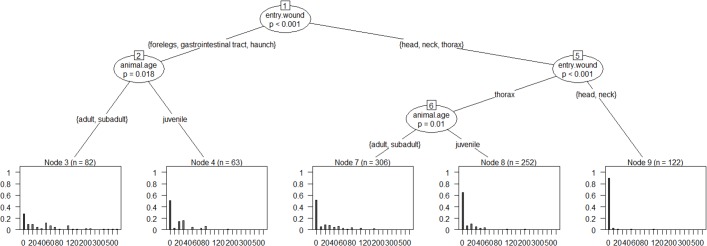
Inference tree for assessment of the factors that influence the escape distance of wild boar (including animals that remained on the spot). The inference tree can be read from top to bottom. The number of the splits (nodes) is shown in the boxes (e.g. 1). Significant variables are indicated at the splits with the significance level in the ellipses. Connections between the nodes show the group distributions. The escape distance is shown as the end node of the inference trees with the help of relative frequencies (with details of the sample size n).

The inference tree in Fig 10 takes account of all data on wild boar. Here, the location of shot placement has a significant (p<0.001) effect on the length of the escape distance (split 1). The escape distance following hits to the thorax, the head or the neck differs from the escape distance with hits to the forelegs, the gastrointestinal tract or the haunch.

In the case of hits to the forelegs, the gastrointestinal tract or the haunch, roughly 30% of the subadult or adult animals remained on the spot, in contrast to 50% of the juvenile animals below one year (split 2, *p* = 0.018). The escape distances also differed when the hit was to the thorax compared to when it was to the head or neck (split 5, *p*<0.001). Around 52% of adult or subadult animals remained on the spot if the hit was to the thorax, compared to roughly 65% of the juveniles (split 6, *p* = 0.01).

#### Analysis of inference trees using hurdle models for wild boar

The individual splits from Fig 10 are analysed in more detail using multivariable hurdle models in Table 2.

**Table 2:** Hurdle model for wild boar; dependent variable: Escape distance.

	Count model- Zero-truncated poisson			Zero hurdle model Binomial		
	β^1^	Standard Error	*p*	β^2^	Standard Error	*p*
**Split 1: Comparison entry wound**						
Forelegs, gastrointestinal tract, haunch (intercept)	4.309	0.091		0.493	0.171	
Thorax, head, neck	-0.432	0.107	***	-1.052	0.189	***
**Split 2: Condition forelegs, gastrointestinal tract, haunch**						
Juvenile (intercept)	3.691	0.166		-0.032	0.252	
Subadult, adult	0.772	0.205	***	0.957	0.353	**
**Split 5: Comparison entry wound**						
Head, neck (intercept)	4.076	0.243		-2.033	0.321	
Thorax	-0.206	0.249	0.41	1.648	0.332	***
**Split 6: Condition thorax**						
Juvenile (intercept)	3.636	0.085		-0.639	0.134	
Subadult, adult	0.353	0.107	***	0.558	0.177	**

^1^ Escape distances of 10 m and more.

^2^ The hurdle part estimates the probability with which the escape distances occur (10 m or more), relative to the probability that no escape distances occur (< 10 m).

** *p*<0.01

*** p<0.001

**Fig 11:**
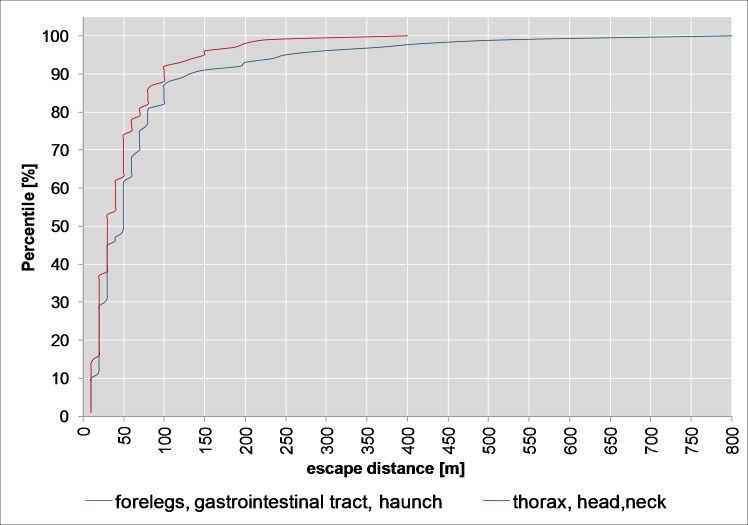
Cumulative density plot for split 1 (wild boar).

The escape distances were shorter (90th percentile approx. 100 m, median 30 m, max. 400 m, Fig 11) with hits to the thorax, head or neck (split 1) than with hits to the forelegs, the gastrointestinal tract or the haunch (90th percentile approx. 132 m, median 50 m, max. 800 m, Fig 11). The difference is significant (count data part, *p*<0.001, Table 2). The percentage of animals with escape distances of 10 m or more is lower (binomial part, *p*<0.001, Table 2) in the case of hits to the thorax, the head or the neck (approx. 36%) than to hits to the forelegs, the gastrointestinal tract or the haunch (62%).

**Fig 12:**
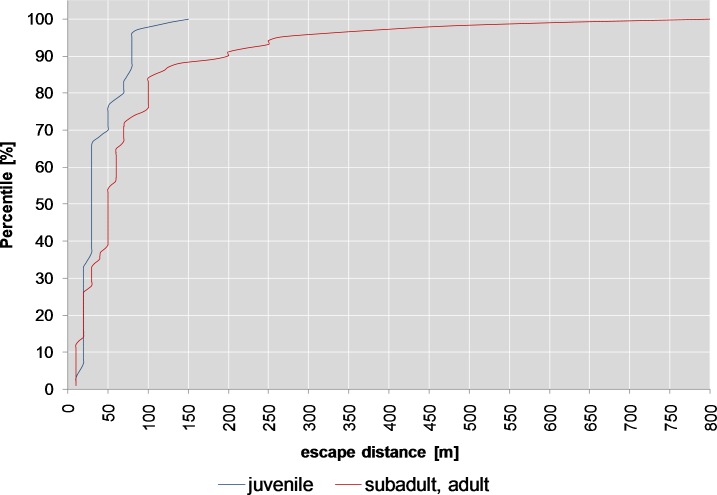
Cumulative density plot for split 2 (wild boar).

When wild boar were hit in the forelegs, the gastrointestinal tract or the haunch (split 2, Table 2), the escape distances (10 m or more) of adult or subadult animals were significantly higher (90th percentile 200 m, median 50 m, max. 800 m, Fig 12, count data part, *p*<0.001) than in juvenile animals (90th percentile 80 m, median 20 m, max. 150 m).

While only around 30% of the adult or subadult animals remained on the spot (escape distance <10 m) this was the case in roughly 50% of juveniles (binomial part, *p*<0.01, Fig 10).

**Fig 13:**
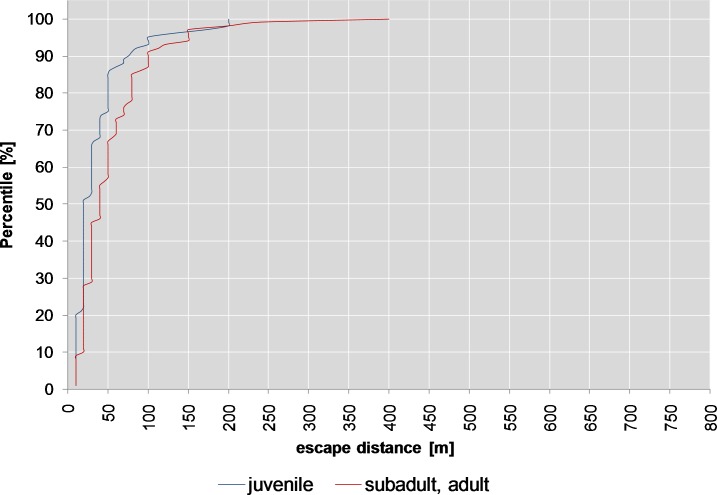
Cumulative density plot for split 6 (wild boar).

When shot in the head or neck, around 90% of the animals remained on the spot (binomial part, *p*<0.001, split 5, Table 2), compared to approx. 58% of animals with hits to the thorax. Around 52% of adult or subadult animals remained on the spot if the hit was to the thorax (binomial part, split 6) compared with roughly 65% of juvenile animals (*p*<0.01, Fig 10, Table 2). There are also significant differences (count data part, p<0.001, Table 2) between the escape distances (10 m or more) of subadult or adult animals (90th percentile 100 m, median 40 m, max 400 m, Fig 13) and juvenile animals (90th percentile 76 m, median 20 m, max 200 m).

Neither the bullet material, the bullet type, the shooting distance nor the sex of wild boar were found to have any influence of the length of the escape distance of wild boar.

## Discussion

Here,—in one of the largest studies so far—escape distances of roe deer and wild boar were compared in order to analyse whether lead or non-lead ammunition showed a significantly different killing efficiency. There was no difference based on bullet material between the percentage of the two wildlife species that had no or only a very short escape distance (<10 m). Moreover, neither was there any significant difference in the average length of the escape distance (10 m or more) between animals shot using lead ammunition and those shot with non-lead bullets. Our research does not suggest that non-lead ammunition leads to an unreliable killing effect as indicated in some previous studies [12, 13]. Other parameters can play a more decisive role.

Lead hunting ammunition can have negative effects for consumers, the game itself and the environment therefore replacing lead ammunition with non-lead ammunition appears recommendable. There has been, however, also criticism of the use of non-lead ammunition. For instance, practical tests using non-lead and lead ammunition variants conducted by the Association of German Professional Hunters (BDB) showed longer escape distances with the non-lead ammunition [12]. What was not taken into account, however, was that there are numerous interacting variables. A simplified comparison of the escape distances of game animals following hits with non-lead or lead ammunition is not to be recommended [15]. We suggest that some of the other studies may not have taken possibly interfering parameters into account.

Other criticism focussed on the fact that there is no satisfactory full range of non-lead bullets for the standard hunting calibres. Only tried-and-tested bullet designs were used in the project. This might explain why there was no difference between non-lead and lead ammunition in terms of killing effect. Likewise, the non-lead bullets considered in the study of Hackländer et al. [15] had been tested at shooting ranges before field use, and these authors also reported that the length of escape distance was not related to the bullet material.

The escape distance is considered to be the decisive, observable and assessable benchmark for evaluation of the effect of the shot following the bullet impact [17].

In the past, there have been at least two methods to classify escape distance. In terminal ballistic study models for hunting ammunition, the escape distances were divided into 2 groups: ≤ 25 m and > 25 m [27]. The reason given by the authors was that escape distances of ≤ 25 m are still acceptable for the hunter and do not necessitate any lengthy searches. We did not use this classification in our analysis, as no information is currently available on the escape distance that can still be considered acceptable. Creating the “<10 meters” category from the groups “0 meters” and “1 m to 9 meters” was supported by the fact that only a few animals died between 1 and 9 meters, while many were reported for “0 meters” and “≥10 meters”.

There has been criticism of the use of the escape distance as a method to determine the killing effect [37]. The key benchmark for the killing effect of a bullet is the amount of energy that is active at a specific point in the body of the game. This energy determines the extent of the injuries. The distance covered by an animal after the shot does not necessarily indicate when the animal actually died. Moreover, the estimates of the length of the escape distance of roe deer and wild boar by the hunters are sometimes very imprecise [37]. However, it would be extremely time-consuming and impracticable to measure the energy released within the body of the animal, therefore escape distance appears to be at the moment a reasonable choice to measure the killing effect [17, 21].

The current study used an up-to-date statistical approach to analyse the data in detail. Conditional regression trees identified possible interactions between different variables, such as bullet material and bullet type, location of shot placement, hunting method, shooting distance, and age and sex of the animals. The analysis elucidated that other factors had a more important influence on the escape distance than the type of bullet material (lead or non-lead). In the case of roe deer, the shot placement had the greatest influence on the escape distance. Hits to the forelegs, the gastrointestinal tract, the haunch or the neck resulted in longer escape distances than hits to the thorax or head. A thorax shot with rapid killing effect and minimal destruction of the game meat, low microbial burdens and a short escape distance following the shot is recommended [38, 39]. Shots to the gastrointestinal area are not acceptable on animal welfare grounds and for reasons of game meat hygiene [39]. Shots to the intestines should also be avoided where possible, as these hits can damage organs that are not directly vital to survival and therefore result in longer escape distances [17]. Neither do shots to the haunch or the forelegs and hind legs result in the fastest possible death of the game, and longer escape distances are generally to be expected in all game types following such hits [17]. Only a thorax hit guarantees rapid death [40]. Shooting distance in combination with the location of the shot placement also had an impact on escape distances.When either the forelegs, the gastrointestinal tract, the haunch or the neck of the animals were hit over longer shooting distances (above 100 m), the percentage of animals with escape distances of ≥10 m was considerably higher than in animals shot over lower shooting distances (60% vs. 30%). Longer escape distances for roe deer and red deer with shooting distances from 100 m have been reported in a previous study [19]. With roe deer, this effect was only observed with copper ammunition, while in the case of red deer the correlation did not depend on the bullet material [19]. Our study was also unable to find any difference between the two bullet materials based on shooting distance but only differences based on the location of shot placement.

The type of hunting–driven hunts, hunting from hides and stalking—also had an influence on escape distances, which intuitively makes sense and is supported by other studies [41]. The percentage of roe deer with escape distances of 10 m or more was higher in driven hunts than with hunting from hides and stalking, but this only applied to shots to the thorax or head. Thorax shots are more easily achievable when hunting from hides than in hunting on the move. The latter involves shooting under time pressure, which means an optimum hit is less likely than from a hide [39].

The 825 records for shooting of wild boar showed that hits to the forelegs, the gastrointestinal tract or the haunch resulted in longer escape distances than hits to the thorax, the neck or the head. Following hits to the forelegs, the gastrointestinal tract or the haunch, approx. 70% of adult and subadult animals and 50% of juveniles had escape distances of ≥10 m. When shot in the thorax only 48% of the adult/subadult animals and 35% of the juvenile animals showed escape distances ≥10 m. The young animals are more likely to die on the spot, because the wound torn by a rifle bullet is larger in relation to the size of the smaller animals’ vital organs than in larger adults. This leads to faster incapacitation times due to quicker oxygen deprivation of the brain.

Field reports from the Association of German Professional Hunters (BDB) indicate doubts over non-lead bullets with regard to humane killing in the case of long shooting distances and heavy game [37]. We were not able to confirm these findings in our work, as the escape distances of wild boar were influenced neither by the shooting distance nor by the bullet material.

The bullet type–such as fragmenting bullets / partially fragmenting bullets or deforming bullets—did not have any influence on the length of the escape distance of roe deer and wild boar. The research project "Safety of game meat obtained through hunting" [13] only used bullet types that were rated as positive by hunters in previous studies (rather than a cross-section of the market). This is confirmed by the statement that newly developed non-lead hunting bullets kill the game in a humane way [42]. This could explain why there is no difference between the escape distances with the use of deformation bullets on the one hand and fragmenting or partially fragmenting bullets on the other.

The bone hits were documented in the shooting records but were excluded from further analysis as there was a high correlation with the shot placement. Bones were hit significantly less frequently when animals were shot in the stomach/intestines (above all in the stomach). Some factors could not be considered in the analysis. Nothing is known regarding the behaviour of the animals prior to the shot being fired (grazing, moving slowly, alarmed and hence stressed). This might also have a major influence on the escape distance. Neither is anything known about the location of the kill (open land, forest). This in turn influences the shooting distance; the maximum shooting distances in a forest are generally lower than on open land [17].

The influence of the hunters skills could also not be taken into consideration. The shooting skills (above all in the twilight hours) would be an important criterion for assessment of the shot placement and therefore of the escape distance of the animals. There are, for example, significant differences between the average daylight conditions for the killing of roe deer on the one hand and wild boar on the other. The light conditions for the hunting of wild boar are generally poorer than for roe deer [42].

The 2,059 evaluated shooting records did not provide any indication that non-lead ammunition results in longer escape distances of roe deer or wild boar. The length of the escape distance depends on other factors, such as shot placement, shooting distance, hunting method or age of the animals.

In future, it will be necessary to denote on ammunition packages the maximum shooting distances for each relevant game species [43]. It was not possible to include the ideal or maximum shooting distance for each bullet type in the analysis during the project "Safety of game meat obtained through hunting", as the necessary information was not available.

The results also confirm the finding of other studies that the bullet material is not the decisive factor in killing effect in the case of roe deer and wild boar [14–18]. The discontinuation of the use of lead ammunition presupposes the existence of alternatives to lead ammunition that permit future hunting activities. Alternatives do in fact exist: in recent years, non-lead bullets have been developed that can replace the lead bullets currently used and that also do not differ from lead bullets in terms of price [44, 45].

## Supporting information

S1 Table Variables and their description, including unit and measurement scales. (PDF)
