# Supplementary material for: Hunting of roe deer and wild boar in Germany: Is non-lead ammunition suitable for hunting?
Source: PLoS One. 2017 Sep 19;12(9):e0185029. doi: 10.1371/journal.pone.0185029 (PMC5605046; doi:10.1371/journal.pone.0185029)
Supplement: S1 Table — (PDF) [file pone.0185029.s001.pdf]

Variable	Description	Unit	Measurement Scales
Escape distance	in 10 m classes, subjective evaluation by hunters	meter	Interval scale
Bullet material	1 non-lead<br>2 lead	-	Dichotomous
Bullet construction	1 mass loss expected in the target media (fragmenting projectiles / partial fragmenting projectiles)<br>2 mass loss not expected in the target media (deformation projectiles)	-	Dichotomous
Location of shoot placement (entry wound)	1 thorax (chest) cavity<br>2 gastrointestinal tract (stomach, gut)<br>3 haunch<br>4 head<br>5 neck<br>6 forelegs	-	Categorial
Hunting method	1 stalking<br>2 hunting from hides<br>3 drive hunting	-	Categorial
Age of game	1 juvenile (<1)<br>2 subadult (1-< 2)<br>3 adult (from 2)	years	Ordinal
Shooting distance	Subjective evaluation by hunters	meter	Continuous
Bone hit	1 no<br>2 yes	-	Dichotomous
Subsequent search	1 no<br>2 yes	-	Dichotomous
Sex of game	1 female<br>2 male	-	Dichotomous
